# Unveiling the Role of Cross-Cultural and Cognitive Differences in Organizational Learning Mechanism of Technology-Acquiring Cross-Border Mergers and Acquisitions: Evidence From Emerging Market Enterprises

**DOI:** 10.3389/fpsyg.2022.863442

**Published:** 2022-05-06

**Authors:** Kanxiang Chen, Xuanmei Cheng, Run Zhang, Giuseppe Cillo, Antonio Ragusa

**Affiliations:** ^1^School of Economics, Zhejiang University of Technology, Hangzhou, China; ^2^School of Management, Zhijiang College, Zhejiang University of Technology, Hangzhou, China; ^3^School of Management, Zhejiang University of Technology, Hangzhou, China; ^4^Department of Agronomy, Animals, Food, Natural Resources and Environment, University of Padova, Padua, Italy; ^5^Rome Business School, Rome Business School Nigeria and Space Economy Institute, Rome, Italy

**Keywords:** cross cultural, cultural differences, cognitive differences, organizational learning, technology-acquiring cross-border mergers and acquisitions, emerging market

## Abstract

In recent years, cross-border mergers and acquisitions (M&As) of emerging market enterprises (EMEs) have increased rapidly, and many cross-border M&A have been conducted in the United States, Western Europe, and other developed countries. This new type of technology-acquiring cross-border M&A has several unique features. In particular, the cross-cultural differences between the home country and the host country and the cognitive differences between emerging markets and developed markets pose a huge challenge to the organizational learning of technology-acquiring cross-border M&A of enterprises from emerging markets. Based on this, the present study innovatively constructs an integrated theoretical model to explore the role of cross-cultural and cognitive differences in the organizational learning mechanism of technology-acquiring cross-border M&A in emerging markets. In this study, the partial least squares structural equation model (PLS-SEM) was used in an empirical study of 240 Chinese technology-acquiring cross-border M&A enterprises, and it was found that cultural and cognitive differences play an important role in technical ability and learning performance. The study also found that the interaction of cross-cultural differences between the home and host countries and the cognitive differences between the emerging and developed markets promoted the learning performance of cross-border M&A in the emerging markets. Based on the integration theory of cultural differences, cognitive differences, and technical ability, this paper unveils the role of cross-cultural and cognitive differences in organizational learning mechanisms of technology-acquiring cross-border M&A.

## Introduction

In recent years, emerging market enterprises (EMEs), represented by China, have developed rapidly and have conducted several technology-acquiring cross-border mergers and acquisitions (M&As) (Kale and Singh, 2016) with enterprises in developed countries. According to data from the United Nations Conference on Trade and Development (UNCTAD) from 2010 to 2020, cross-border M&A from Chinese enterprises have increased rapidly in recent years; in particular, technology-acquiring cross-border M&A with the United States, Western Europe, and other developed countries have increased significantly.

What factors affect the learning performance of technology-acquiring cross-border M&A of EMEs? Many scholars have conducted in-depth studies on this topic in an attempt to open the black box of technology-acquiring cross-border M&A learning mechanisms of EMEs (Levitt and March, 1988; Aulakh, 2007; Chen et al., 2012). Existing studies are mainly based on the relevant factors of technical capability (Bertrand and Capron, 2014; Bhaumik et al., 2015), such as absorptive capacity (Deng, 2010; Bilgili et al., 2016; Zhou et al., 2018; Duan et al., 2021), knowledge scale (Farnese et al., 2019), technological gap (Bonaglia et al., 2007), R&D investment (Chen et al., 2012), and other factors that affect cross-border knowledge transfer (Ai and Tan, 2018; Haasis et al., 2018; Hernandez and Guillén, 2018). Existing studies have mainly emphasized the impact of a single factor or a few factors of technical capability (Buckley and Hashai, 2014; Bhaumik et al., 2015) on the learning performance of technology-acquiring cross-border M&A, which limits the research results (Gomes et al., 2012).

In fact, the technology-acquiring cross-border M&A initiated by EMEs have many unique features (Madhok and Keyhani, 2012), especially in the learning process of reverse technology-acquiring cross-border M&A (Liu and Meyer, 2020) with developed markets, the cultural differences between home countries and host countries (Buckley et al., 2007, 2009; Dikova and Sahib, 2013; Cui et al., 2016), and the cognitive differences between emerging and developed market enterprises. This poses a great challenge to the organizational learning of the technology-acquiring cross-border M&A of EMEs. Cross-cultural effects, including psychological distance (Masuda et al., 2020), the liability of foreignness (Dikova and Sahib, 2013; Sachsenmaier and Guo, 2019), and cross-cultural and cognitive differences, have an important impact on the knowledge transfer and organizational learning of M&A enterprises. The organizational learning of technology-acquiring cross-border M&A of EMEs is a complex internal learning process (Graebner et al., 2017; Mathews, 2017), and its learning efficiency is influenced by multiple situational factors, such as cross-cultural differences (Dikova and Sahib, 2013; Nicholson and Salaber, 2013), cognitive differences, technological capability, and others. Technological capability is the basis of technology acquisition. In the complex situation of technology-acquiring cross-border M&A of EMEs, the complex synergistic effect of cultural differences and cognitive differences on technological capability becomes more prominent. Among them, cultural difference emphasizes external representation of the environment between the home country and the host country, especially in supervision and culture (Bauer et al., 2014), while cognitive difference emphasizes internal representation of the environment, rooted in the mind (Xu and Shenkar, 2002).

To sum up, this study innovatively constructs an integrated theoretical model to explore the role of cross-cultural and cognitive differences in organizational learning mechanisms based on the technical capabilities of technology-acquiring cross-border M&A in emerging markets and considers how EMEs can overcome the barrier of cultural differences between the home country and the host country as well as the limitations of cognitive differences between the emerging and developed markets, in an attempt to open the black box of the mechanism of technology acquisition in the cross-border M&A of EMEs.

## Theory and Hypothesis Development

### Literature Review

#### Organizational Learning Mechanism of Technology-Acquiring Cross-Border M&A

Research on organizational learning has long been a core part of international business and strategic management research. Taking the Uppsala internationalization model as an example, the knowledge acquired by the international business of EMEs from home and host countries has been an important source of competitiveness for internationalization (Johanson and Vahlne, 1977; Wiedersheim-Paul et al., 1978). Johanson and Vahlne (2009) revised the model to include the effect of non-empirical knowledge from other companies in the internationalization process. The model suggests that a firm’s international expansion is a function of its knowledge learning, which can be gathered from its business in the market or from the inter-firm network in which the firm participates (Elango and Pattnaik, 2007; Chin et al., 2021b). The model emphasized the lack of knowledge as a barrier to internationalization as it increases its perceived risks and costs. This model captures both the evolutionary and behavioral dimensions of enterprise internationalization, highlighting how enterprises source knowledge in the process of internationalization (Andersson et al., 2002; Johanson and Vahlne, 2009).

Mergers and acquisitions are regarded as ways to absorb technological knowledge, especially for EMEs. As a result, they have become an important method for EMEs to obtain external knowledge. Numerous studies have shown that the internationalization of EMEs is often based more on technology utilization than technology development (Lall, 2000; Gubbi et al., 2010). Purchasing know-how helps EMEs fill in their know-how gaps, catches up with peers, and improves their own technology capabilities by combining purchasing know-how with internal R&D (Dunning and Lundan, 2008). Some studies (Cassiman and Veugelers, 2006; Buckley et al., 2016) also showed that knowledge complimentarily enhanced the competitive advantage of EMEs, which had a positive effect on the internationalization of enterprises.

#### Cross-Cultural Differences Between Home and Host Countries and the Organizational Learning of EMEs From Cross-Border M&A of Technology-Acquiring Cross-Border M&A

Culture is regarded as a key factor in determining the structure and behavior of enterprises (Powell, 1983; Shenkar, 2001; Stahl and Voight, 2004; Zhao and Zhang, 2005; Chakrabarti et al., 2009; Vrontis and Dauber, 2012). Significant cultural differences trigger the conflicts (Sarala, 2010) between the demands for external legitimacy (or local responsiveness) and internal coherence (or global integration) of the multinational enterprise system. Cultural differences are one of the most frequently examined constructs in the international business literature (Stahl K. and Voigt A., 2005; Bouwman, 2013), and balancing these conflicting results has been a key challenge for multinational enterprises. Shenkar (2001) points out that few concepts are more widely accepted in international business studies than cultural differences. Cho and Padmanabhan (2005) propose that, generally, studies of international business cannot be conducted unless they include a clear variable controlling for cultural differences. Finally, Zaheer and Nachum (2012) argue that “international management is difference management in essence.” Earlier research has found that firms are less likely to invest directly in markets with distant cultural differences (Björkman et al., 2007).

Multinational enterprises operating across the countries face at least two different institutional cultures, namely those of the home and host countries. Cultural differences refer to the degree of cultural differences between the home and host countries. Kostova and Zaheer (1999) further propose that the greater the cultural difference, the more difficult it is for multinational enterprises to establish legitimacy in host countries and transfer the strategic practices of the home country to foreign subsidiaries (Kostova, 1999). Therefore, cultural differences between the home country and the host country are often analyzed in terms of supervision and culture (Bauer et al., 2016). Enterprises implementing cross-border M&A are faced with cross-cultural differences between the home and host countries, and they should try their best to narrow these differences through technology-acquiring M&A (Stahl G. K. and Voigt A., 2005). First, home country enterprises need to fully understand the host country’s market rules and other aspects of culture (Zhu and Huang, 2007; Weber and Drori, 2008; Denison et al., 2011); second, home country enterprises need the host country’s corporate culture system to fully recognize their organizational culture (Sarala, 2010; Weber and Drori, 2011). Therefore, the organizational learning of technology-acquiring M&A of home country enterprises must overcome cultural differences. This process is essentially an organizational learning process (Popli et al., 2016).

#### Cognitive Differences Between Emerging and Developed Market Enterprises and Organizational Learning of Technology-Acquiring Cross-Border M&A

Cognition emphasizes the actor’s internal representation of the environment rooted in cultural orthodoxy (Xu and Shenkar, 2002). Abdi and Aulakh (2012) argue that inter-firm relations between partners from cognitively distant environments are affected by governance difficulties due to the lack of a common cognitive framework. Some scholars have studied the cognitive differences in cross-border M&A enterprises from the cognitive and social norms of their home countries and host countries (Bhal et al., 2009; Skvortsova and Vershinina, 2021).

In most cross-border M&A, the integration of post-M&A is a potentially significant challenge and may be difficult to achieve between companies in different countries; therefore, the challenge of organizational learning performance is often affected by cognitive differences between enterprises in emerging and developed markets (Rui and Lan, 2011). The organizational learning of cross-border M&A of EMEs often involves double-layered acculturation or assimilation (Barkema et al., 1996). Nahavandi and Malekzadeh (1988) point out the impact of cognitive differences on M&A integration. Closely related to this concept, Chatterjee et al. (1992) propose that the degree of cognitive differences negatively affects a shareholder’s value when strong integration is required (Chin et al., 2021a). Weber et al. (1996) have built their research on a theoretical framework based on cognitive differences and potential adaptation of different characteristics and found that corporate cognitive differences affect the cooperation between the executives of two companies; when corporate cognition has significant differences, both sides have a negative attitude toward cooperation. Larsson and Finkelstein (1999) point out that negative attitudes, at the employee level, play an important role in the performance of organizational learning in M&A. Employee resistance refers to the positive or negative opposition attitude from a single employee or collective of employees toward the M&A transaction, which is negatively related to the success of M&A. Related studies on cognitive differences that negatively affect the performance of cross-border M&A include lack of communication (Schweiger and Denisi, 1991), neglect of human nature (Buono et al., 1989), and stress level (Cartwright and Cooper, 1993).

Therefore, the organizational learning of technology-acquiring cross-border M&A of EMEs needs to integrate cross-cultural differences, cognitive differences, and learning mechanisms to form an integrated theoretical framework. The role of cross-cultural and cognitive differences in the organizational learning mechanism of technology-acquiring cross-border M&A in the emerging markets is a black box of the technology acquisition of EMEs’ cross-border M&A. Technological capability is the basis of technology acquisition, while cultural and cognitive differences have the external and internal moderation on technological capability. In this study, we aim to combine the interaction of many influencing factors to explore this learning mechanism, concluding that it is based on the technical capability of multinational EMEs (Buckley, 2009; Buckley and Strange, 2011) and moderated by cross-cultural and cognitive differences.

We attempt to construct an integrated theoretical model. First, we aim to understand the learning mechanism of cross-border M&A based on technical capabilities. Second, and most importantly, we need to explore the core role of cross-cultural and cognitive differences between the two sides, which have an important moderating effect on the learning mechanism of EMEs. Under the interactive effect, this study proposes hypotheses on the interactive relationship between the learning performance of technology-acquiring cross-border M&A of EMEs, the technical capabilities of enterprises, and the cross-cultural and cognitive differences of both sides of M&A, as shown in Figure l.

**FIGURE 1:**
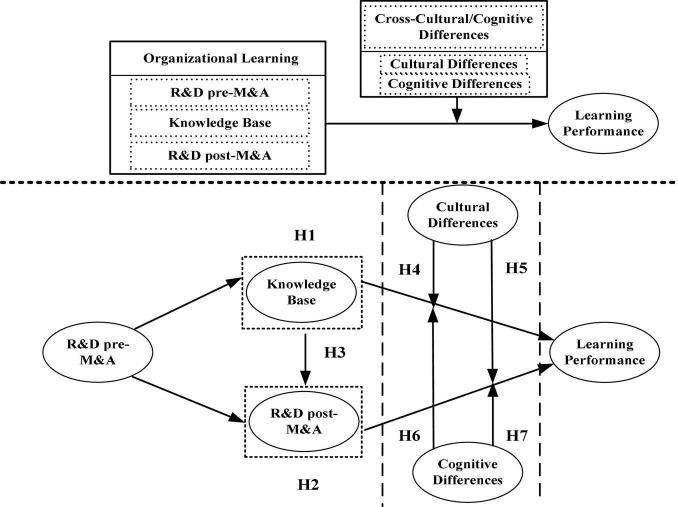
The theoretical model of organizational learning mechanism research of technology-acquiring cross-border M&A of EMEs.

### Hypothesis Development

#### Organizational Learning Mechanism Based on the Technical Capability of Technology-Acquiring Cross-Border M&A

##### Learning Mechanism 1: The Mediating Effect of Knowledge Base Between R&D Intensity of Pre-M&A and Learning Performance

Previous studies show that a certain relationship exists between technology acquisition in cross-border M&A and technology capability. The technological ability of an enterprise affects its performance by acquiring technology (Fink, 1998). To imitate and improve the existing technology and create new products, M&A enterprises need to have a certain skill level and ability. Pre-M&A R&D investment can help enterprises accumulate their knowledge base, thus promoting the improvement of their technology. Post-M&A learning must imitate and add some modifications to the accumulated knowledge base and then create new products, so as to improve the performance of an enterprise in acquiring technology through cross-border M&A.

Through cross-border M&A, enterprises must also absorb the information and technical know-how of acquired enterprises in the developed countries (Ahuja, 2000; Powell et al., 2005). For an acquiring enterprise, it should not only be understood from the explicit aspect of its knowledge but it should also take into account the practice and implicit knowledge embedded in the knowledge base. The knowledge-based theory proposes that knowledge can be conceptualized along a continuum that includes tacit knowledge acquired through experience and embedded in individual cognition and organizational conventions and knowledge embodied in specific products and processes (Inkpen and Dinur, 1998). The central challenge facing an organization is transforming tacit and embedded knowledge into explicit and concrete knowledge. This transformation is possible by creating capabilities and conventional forms of knowledge about how to perform productive tasks (Langlois and Robertson, 1993). One of the key decisions that managers make when looking for complementary knowledge pools is to assess the extent to which the acquired knowledge base can be absorbed, integrated, diffused, and deployed and to use it within the organization to benefit from it (Madhok, 1996). While knowledge can be embodied in certain products, technologies, or services, Madhok (1996) emphasizes that an equally important and valuable aspect of knowledge is the informal component that appears in the form of basic functions and conventions that support the final product. Practice is considered a unique form of knowledge base that can only be learned by doing (Felin and Hesterly, 2007). Therefore, R&D investment pre-M&A can help enterprises accumulate explicit and implicit knowledge bases; enhance the capability of absorption, integration, diffusion, and innovation; and thus improve the performance of acquiring technologies in cross-border M&A.

Knowledge base appears to play an intermediary effect between R&D intensity of pre-M&A and enterprise learning performance. Therefore, this study proposes the following hypothesis:

*Hypothesis 1: Knowledge base has a mediating effect on the relationship between R&D intensity of pre-M&A and enterprise learning performance.*

##### Learning Mechanism 2: The Mediating Effect of R&D Intensity Post-M&A Between R&D Intensity Pre-M&A and Learning Performance

Since M&A enterprises in developed countries can absorb and utilize external knowledge (Cohen and Levin, 1989; Roller and Waverman, 2001), in the case of R&D economies of scale, enterprises are more likely to improve their efficiency after M&A, and this technology transfer process will encourage them to increase the R&D investment of post-M&A. Further, acquiring enterprises that create authorization based on capability will increase R&D investment in post-M&A as the addition of complementary R&D facilities helps to develop new capabilities within multinational enterprises. In addition, if the enterprise operates globally, the development of new capabilities and the transfer of resources will be improved, and enhanced R&D intensity will have a positive effect on product quality and product line expansion (James et al., 1998; DeYoung et al., 2009).

Nevertheless, to improve the existing technologies, latecomers enhance the R&D intensity of post-M&A by cooperating with foreign partners in the developed countries in various ways to absorb more information and know-how (Pack and Westphal, 1986; Lall, 1993; Kim, 1999; Ahuja, 2000; Powell and Grodal, 2005). Acquiring enterprises often conduct joint production and development with acquired enterprises by enhancing the R&D intensity of post-M&A to promote the learning performance of technology acquisition; thus, co-production and co-development are promoted (Kim, 1999). For example, developed countries often restrict the export of cutting-edge technologies due to their strategic considerations or to maintain their technological edge (Lee, 2008). Co-production means that the latecomer retains to produce copies of the complete product or critical parts, which may provide them with independent production capacity. Co-development refers to the collaboration during the development and design phase as well as the acquisition of design and system integration expertise by developers in the later phase. Scholars have generally stressed the importance of acquiring technology from foreign partners through technology licensing, joint development, and joint research agreements.

It can be seen that the R&D intensity of post-M&A has an intermediary effect on the relationship between R&D intensity of pre-M&A and learning performance. Therefore, this study proposes the following hypothesis:

*Hypothesis 2: The R&D intensity of post-M&A has a mediating effect on the relationship between the R&D intensity of pre-M&A and enterprise learning performance.*

##### Learning Mechanism 3: The Sequential Mediating Effect of Knowledge Base and R&D Intensity of Post-M&A Between R&D Intensity of Pre-M&A and Learning Performance

Increasing international competition and rapid technological changes have pushed firms to maintain their competitive positions. In this context, technical expertise, market knowledge, flexibility, and innovation capability have become key assets for enterprises (Cantwell and Santangelo, 2003). The technical capabilities of an enterprise, such as skills, knowledge, connections, implicit components, and conventions, are the key resources for generating and managing technological changes (Piscitello, 2004). M&A is regarded as an important means for merging enterprises to absorb their respective technologies. This process means that enterprises acquire knowledge assets quickly through cross-border M&A, which is an important way to increase the knowledge base and facilitate greater development of technologies available to acquired enterprises (Granstrand et al., 1992). After M&A, the absorption and utilization of external knowledge promote the improvement of technical capabilities (Cohen and Levin, 1989; Roller and Waverman, 2001), which encourages enterprises to make more internal efforts and R&D investments to take advantage of new technologies. Therefore, cross-border M&A promotes an increase in the knowledge base, which further promotes M&A to increase the R&D intensity of post-M&A in order to use new technologies; this will have a positive effect on the company’s innovation capability, especially in terms of innovation output (Cockburn and Henderson, 1996).

Different types of knowledge base between the acquiring enterprise and the acquired enterprise will also lead to an increased R&D investment of the acquiring firm post-M&A. After M&A, the absorption and utilization of external knowledge will lead to an improvement in the knowledge base (Cohen and Levin, 1989; Roller and Waverman, 2001). When the knowledge bases of the two companies are quite different, the application of new knowledge may require the introduction of several changes within the organization, resulting in the interruption of organizational routines, which will further increase the R&D intensity of the enterprise post-M&A. Therefore, the improvement of technology transfer and knowledge base will encourage enterprises to increase their R&D investment of post-M&A, so as to make use of the acquired new technology and have a positive effect on the innovation ability of the company, especially in terms of learning performance (Cockburn and Henderson, 1996).

It can be concluded that the knowledge base and R&D intensity of post-M&A have a successively mediating effect on the R&D intensity of pre-M&A and enterprise learning performance. Therefore, this study proposes the following hypothesis:

*Hypothesis 3: A knowledge base and the R&D intensity of post-M&A successively mediate the relationship between R&D intensity of pre-M&A and enterprise learning performance.*

#### Moderating Effect of Cultural Differences Between Home and Host Countries in the Organizational Learning Mechanism

North (1990) proposes that organizations are purposeful entities designed by their creators to maximize wealth, income, or other goals defined by the opportunities provided by sociocultural structures (North, 1990). The information and knowledge required by an organization are determined by the specific cultural context because the firm’s profit-maximization behavior can take the form of choice within the existing institutional constraints (North, 1990). On the contrary, an examination of the cultural environment largely gives an impression of the need for different types of knowledge and skills. Therefore, different cultural environment characteristics have different incentives and constraints on tacit knowledge acquisition and skill development (North, 1990).

In the context of M&A, cultural differences between the home and host countries have a significant impact on the acquisition of organizational tacit knowledge and the performance of organizational learning in the acquisition and integration stages. When the level of cultural difference between the M&A country and the host country is high, that is, the cultural difference between the home country of the acquiring enterprise and the host country of the acquired enterprise is significant, it often increases the difficulty of communication between both sides of the M&A in the process of acquiring the technology. Therefore, when the acquiring enterprise obtains advanced technology and other related tacit knowledge, it is often constrained by the strict knowledge management system of the host country, which will make it more difficult to break through the constraints of the relevant management systems. At this point, the stronger the knowledge base of the M&A enterprise, the better is the acquisition of advanced technology and learning performance of EMEs through M&A. In other words, the greater the cultural difference between the home and host countries, the greater is the influence of knowledge base on the learning performance acquired by enterprises through organizational learning. Therefore, this study proposes the following hypothesis:

*Hypothesis 4: Cultural differences between the home country and host country positively moderate the effect of knowledge base and enterprise learning performance such that the positive effect is stronger when cultural differences are low rather than high.*

The integration process of cross-border M&A is influenced by cultural differences between the host country and the home country—mainly cultural intervention and industrial restrictions (Bittlingmayer and Hazlett, 2000). When the degree of cultural difference between the home country and the host country of the M&A company is high, the complexity of M&A integration increases significantly; when the difference is small, it is usually easier to understand and adapt to the cultural environment of the host country (Kostova and Zaheer, 1999). When the difference is too large, M&A enterprises cannot easily understand the culture of the host country’s rules, and cultural differences with the host country may also hinder the M&A integration process, or more time may be needed to invest in completing M&A integration. In addition, the complexity of cultural differences may increase the learning cost and expense of M&A enterprises for the following reasons: first, the cultural differences between both sides are more likely to increase the difficulty and possibility of cross-border M&A learning for EMEs; second, the cultural differences between both sides may lead to a longer time for learning and integrating cross-border M&A; third, the cultural difference between both sides leads to additional learning costs for the acquiring enterprise, which needs to further increase the R&D investment post-M&A to better absorb and learn the knowledge of the acquired enterprise. In the case of significant cultural differences between the home and host countries, the complexity of cross-border M&A integration may increase; therefore, M&A enterprises need to further increase the R&D intensity of post-M&A in the process of integration to achieve the learning performance of cross-border M&A. In conclusion, the greater the cultural difference between the home country and the host country, the more the M&A enterprise will increase its R&D intensity after cross-border M&A, so as to promote the increase of the learning performance of the enterprise after the M&A. Therefore, this study proposes the following hypothesis:

*Hypothesis 5: The cultural difference between the home country and host country positively moderates the effect of R&D intensity and learning performance post-M&A such that the positive effect is stronger when cultural differences are low rather than high.*

#### Moderating Effect of Cognitive Differences Between Emerging and Developed Markets in the Organizational Learning Mechanism

In addition to the cultural differences between the home and host countries of M&A, the cognitive differences between enterprises in emerging markets and those in developed markets should also be considered in the complex learning process of cross-border M&A. Such informal constraints on organizational behavior include reputation, widely accepted standards of behavior, and social conventions. However, the constraints of cognitive differences, such as cultural differences, are country-specific. Therefore, it is challenging for the emerging markets to learn from the integration of cross-border M&A in the developed markets because the impact of cognitive differences in solving incompatibility problems is huge (Weber et al., 1996; Very et al., 1997; Morosini et al., 1998).

Narrowing the knowledge gap between the merging and merged enterprises is a key motivation for EMEs to acquire enterprises from developed countries (Petersen et al., 2008). Cognitive differences have a significant impact on the integrated learning performance of EMEs in cross-border M&A (Kogut and Singh, 1988; Calori et al., 1994; Very et al., 1997; Morosini et al., 1998; Teerikangas and Very, 2006). When merging enterprises try to determine the goals, processes, and operations of the target company, friction should be predicted if the perceptions of both sides are vastly different (Shenkar, 2001). In the practice of cross-border M&A for knowledge-intensive industries that target technology acquisition, the value of knowledge assets is not as easy to determine as the value of tangible assets because (1) the value of knowledge assets is more difficult to observe or measure, and (2) the buyer cannot easily determine which knowledge assets can be transferred (Coff, 1999). Compared to enterprises with similar cognitive values, since M&A enterprises must face integration differences post-M&A, including cognitive adaptation pressure (Very et al., 1997) or cognitive convention difference (Morosini et al., 1998), two companies with cognitive backgrounds from different countries may encounter greater difficulties in evaluating and integrating knowledge assets. With an increase in cognitive differences, the difficulty of integration further intensifies; therefore, the greater the cognitive difference between emerging markets and developed markets, the more likely the value of knowledge to be misunderstood, and the greater the difference and difficulty of integration after the acquisition, the worse the learning performance after acquisition. Conversely, the smaller the cognitive difference, the better the influence of the knowledge base of merging enterprises on the learning performance of enterprises. Therefore, this study proposes the following hypothesis:

*Hypothesis 6: Cognitive differences between emerging and developed markets negatively moderate the effect of the knowledge base and learning performance of the firms such that the negative effect is stronger when cognitive differences are low rather than high.*

The cognition of enterprises in the emerging markets is usually rooted in the general values and cognitive psychology of the host country (Calori et al., 1994). This suggests that these cognitive factors may be inertial and difficult to change, which means that determining how to integrate acquiring strategic resources of enterprises for the most effective use can be even more demanding (Meyer and Altenborg, 2008). During the integration phase, it may be difficult to specify how much knowledge is transferred and whether it can be deployed in a new environment (Haspeslagh and Jemison, 1991). In this case, the degree of cognitive communication and mutual trust between enterprises in the emerging markets and developed countries may be crucial because it may affect the reliability of the information provided by target managers (Very and Schweiger, 2001), and trust is largely determined by the cognitive level of both sides. For example, the cognitive psychology rooted in values is crucial in the integration stage, which directly affects the quantity and quality of strategic resources acquired by the merging enterprise from the target enterprise. The trust level and cognitive difference between both sides may shorten the time to establish effective communication with the target enterprise and plan integration and a strategy shift. Determining key performance indicators (KPIs) or dealing with local antitrust requirements in institutionally different contexts can effectively moderate the causal effect between R&D intensity and actual learning performance post-M&A as they may lead to the achievement or failure of technology acquisition goals. Therefore, the cognitive difference between emerging markets and developed markets should have a negative moderating effect on the relationship between R&D intensity and learning performance post-M&A: the smaller the cognitive difference, the more significant the positive impact of R&D intensity on learning performance post-M&A. Thus, this study proposes the following hypothesis:

*Hypothesis 7: The cognitive difference between emerging markets and developed markets negatively moderates the effect of R&D intensity and learning performance post-M&A such that the negative effect is stronger when cognitive differences are low rather than high.*

## Research Design

The organizational learning of technology-acquiring cross-border M&A of EMEs needs to integrate cross-cultural differences, cognitive differences, and learning mechanisms to form an integrated theoretical framework. In this study, the partial least squares structural equation model (PLS-SEM) was used to unveil the role of cross-cultural and cognitive differences in the organizational learning mechanism of technology-acquiring cross-border M&A.

### Research Method

In this study, a PLS-SEM was used to empirically test relevant theoretical assumptions; the partial least squares method was adopted because, compared with other structural equation models, it is more suitable for various reasons: (1) small or medium number of samples were studied because PLS-SEM has no strict requirements on sample size and residual distribution; (2) testing complex models has many obvious advantages and performances; (3) PLS-SEM is applicable to various types of data structures, including irregular or even related data and variables, and it has no strict settings and requirements for variable distribution.

### Sample Source

This study selected cross-border M&A cases of Chinese companies from the BVD-Zephyr global M&A transaction analysis database from 2000 to 2016 as analysis samples. The BVD-Zephyr statistical database contains M&A data and cross-border M&A data collected from all over the world and is currently one of the main databases to study cross-border M&A. In addition, the relevant data on learning performance and technical ability involved in this study, including patent data, R&D investment, and operating income of M&A enterprises, were all from the CSMAR database. The relevant data on cultural differences were derived from Hofstede’s six-dimensional cultural comparison model, and the data on cognitive differences were derived from the Worldwide Governance Indicators (WGIs) developed by the World Bank. After removing the company samples with serious missing data, we finally obtained 240 M&A events of 108 sample enterprises. From the perspective of industry distribution, of the 108 sample enterprises, 11 belong to the energy industry, 18 to the electrical machinery and equipment manufacturing industry, eight to the electronics industry, 14 to the communication and computer industry, 36 to the transportation equipment manufacturing industry, 18 to the equipment manufacturing industry, and 3 to the pharmaceutical manufacturing industry. These enterprises belong to capital and technology intensive or high-tech industries and may have strong characteristics of technology acquisition.

### Measures

The dependent variable—post-M&A learning performance of the acquiring firm—measured the learning performance of the acquiring firm. We measured patents*_*it*_* as the number of patents granted to acquiring firm *i* in year *t.* Learning performance was based on the number of patents of the acquiring firm obtained 1–4 years after the M&A. We measured the patents granted using three items: invention patents granted, utility model patents granted, and appearance patents granted. Let us consider Ningbo Huaxiang Electronic Co., Ltd. as an example. In 2007, Huaxiang acquired undisclosed parts of a German car from Germany. We measured the number of patents granted by Huaxiang from 2008 to 2011; these patents included invention, utility model, and appearance patents granted.

#### Cultural Differences

Cultural differences refer to the differences in social culture, rules, and regulations between home and host countries. Referring to the research by Kaufmann (2007), we cited the Global Governance Index (WGI) developed by the World Bank. The three subdimensions of political stability and vitality, rule of law, and regulatory quality were used as proxy variables to measure the level of cultural differences among countries. We used Kogut and Singh’s measurement formula (Kogut and Singh, 1988) to construct the cultural differences between host and home countries:


(1) $$C\,D_{j\,k}=L\,n\,\left\{\left(D_{i\,j}-D_{j\,k}\right)/V_{i}\right\}$$

where *j* and k represent the host and home country, respectively; *D*_*ij*_ and *D*_*jk*_ represent the quantitative eigenvalues of a certain dimension of the host and home country, respectively, and *Vi* represents the variance of a dimension. Taking Ningbo Huaxiang Electronic Co., Ltd. as an example, we used the Kogut and Singh (1988) modified index to calculate the three items of the cultural differences between China and Germany by citing the three subdimensions of political stability and vitality, rule of law, and regulatory quality.

Cognitive differences refer to the normative and cognitive differences between emerging markets and developed markets, such as ideology and norms of behavior. We employed the relevant dimension of Hofstede’s distance index to measure these cognitive differences and used the three subdimensions of power distance, uncertainty avoidance, and long-term orientation as proxy variables reflecting cognitive differences in national ideology and norms of behavior. We used Kogut and Singh (1988) measurement formula to construct the cognitive difference between the emerging and developed markets:


(2) $$C\,D_{j\,k}=L\,n\,\left\{\left(D_{i\,j}-D_{j\,k}\right)/V_{i}\right\}$$

where *j* and *k* represent the host and home country, respectively, *D*_*ij*_ and *D*_*jk*_ represent the quantitative eigenvalues of a certain dimension of the host and home countries, respectively; and *V*_*i*_ represents the variance of a dimension. Taking Ningbo Huaxiang Electronic Co., Ltd. as an example, we used the Kogut and Singh (1988) modified index to calculate the three items of the cognitive difference between China and Germany by citing the three subdimensions of power distance, uncertainty avoidance, and long-term orientation.

#### Knowledge Base

The merging knowledge base of a firm refers to the stock and structure of knowledge technology owned by the merging enterprises pre-M&A. By referring to Ahuja and Katila’s measurement of the knowledge base (Ahuja and Katila, 2001), in this study, the numbers of invention patents granted, utility model patents granted, and appearance patents granted and owned by merging enterprises pre-M&A were used as proxy variables to measure the knowledge base of merging enterprises (Cloodt et al., 2006; Wen and Liu, 2011). This study used the total number of patents obtained by the merging firms in the first 3 years of the sample investigation period, pre-M&A, as the knowledge base of the merging firms. These patents included invention, utility model, and appearance patents granted. Taking Ningbo Huaxiang Electronic Co., Ltd. as an example, we measured the number of patents granted to Ningbo Huaxiang from 2004 to 2006. These granted patents include invention patents, utility model patents, and the appearance of patents.

#### R&D Intensity

The R&D intensity of an M&A enterprise refers to the ratio of R&D investment to the total assets or operating income of merging enterprises. Referring to the definitions by Ahuja and Katila (2001), in this study, the ratio of the average annual R&D investment to the average annual total assets and the ratio of the average annual R&D investment to the average annual operating income of the M&A enterprises were used as two test items to measure the R&D intensity, which was during 1–3 years before or after M&A. The two measurement items of R&D intensity pre-M&A were, respectively, the ratio of the average R&D investment in the year pre-M&A to the average annual total asset value and the ratio of the average R&D investment in the year pre-M&A to the average annual operating income value. The two measurement items of R&D intensity post-M&A refer to the ratio of the mean R&D investment in the year post-M&A to the average annual total assets and the ratio of the mean R&D investment in the year post-M&A to the average annual operating revenue, respectively. Taking Ningbo Huaxiang Electronic Co., Ltd. as an example, we measured the ratio of the average R&D investment to the average annual total asset value during 2004–2006 and the ratio of the average R&D investment to the average annual operating income value during 2004–2006 to measure the two items of R&D intensity pre-M&A. Similarly, we measured the two items of R&D intensity post-M&A during 2008–2010.

## Data Analysis and Results

To test the influence of the following variables, including R&D intensity pre-M&A, knowledge base, R&D intensity post-M&A, cultural differences, cognitive differences, and others, on learning performance, a research model was set up to test the path coefficient of the relationship between various variables in the theoretical hypothesis, the interpretation degree *R*^2^ of the model, and the significance analysis of *t*-value through PLS-SEM estimation.

The mediation effect was determined by calculating whether the indirect effect was significant. As bootstrapping is better than the Sobel test in statistical testing, we used a three-step process to test the mediating effect. Specifically, (1) we checked whether the indirect effect of a*b was significant; if yes, there was a mediation effect; and if no, there was no mediation effect; (2) we checked whether the direct effect c was significant; if no, it meant full intermediary; if yes, further observation was needed; and finally, we checked (3) whether a* b*c was positive; if so, the mediation effect was complementary; if not, it was a competitive mediator.

### Hypothesis Test: Moderating Effect of Cultural and Cognitive Differences in Learning Mechanism 1

To test the effect of R&D intensity pre-M&A on learning performance, the intermediary effect of the knowledge base between them and the moderating effect of cultural differences and cognitive differences were examined. In this study, a model was established according to the theoretical hypothesis. Through PLS-SEM estimation, the path coefficient of the relationship between various variables in the theoretical hypothesis, the interpretation degree *R*^2^ of the model, and the significance analysis of the *t*-value were tested.

#### Mediating Effects of the Knowledge Base

**FIGURE 2:**
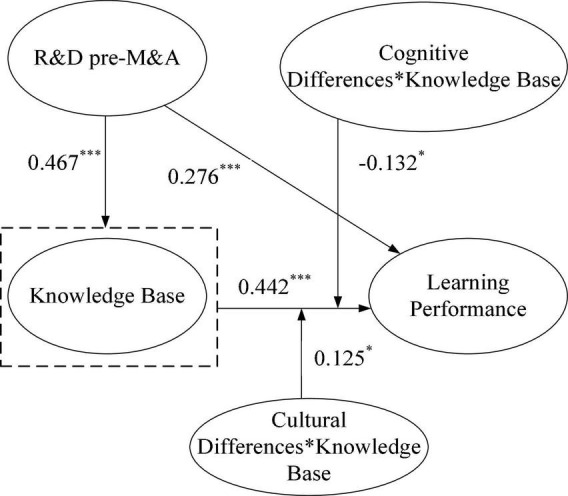
Path analysis results of learning mechanism 1. *p < 0.05 (two-tailed); ***p < 0.01 (two-tailed).

Figure 2 reports the estimated results of Learning Mechanism 1. According to the analysis method of the mediation effect, this should be judged by calculating whether the indirect effect is significant. As bootstrapping is better than the Sobel test for statistical detection, we tested the three-step process of the intermediary effect test. Specifically, (1) we checked whether the indirect effect a (coefficient of PRDwheth* b (coefficient of KB the indirect effect a (coefficient of PRDwhether the indirect effect is significant. As bootstr; (2) we checked whether the direct effect of c (coefficient of PRD→LP) was significant; if no, it meant full mediation; if yes, further observation was needed; and (3) we checked whether a (coefficient of PRD→KB) * b (coefficient of KB→LP) * c (coefficient of PRDLP) was significant; if no, it meant full mediation; if mplementary; if not, it was competitive.

Following a three-step process, we tested the mediating effect and its type. First, we checked whether the significance analysis of the indirect effect (a*b) showed a mediating effect. As shown in Table 1, the R&D intensity pre-M&A has a significant impact on knowledge base (PRD→KB coefficient a = 0.467, *p* < 0.001), and knowledge base has a significant impact on learning performance (KB→LP coefficient b = 0.442, *p* < 0.001). The indirect effect of a*b = 0.467*0.442 = 0.206 (*p* < 0.001) was significant, indicating a mediating effect. Second, after confirming the mediating effect, we checked if the direct effect (c) was significant to judge whether the mediation was full. As shown in Table 1, the R&D intensity pre-M&A has a significant impact on the learning performance of merging enterprises (PRD→LP coefficient c = 0.276, *p* < 0.001), indicating that R&D post-M&A is a partial intermediary for the knowledge base. Finally, we checked whether a*b*c was positive when the direct effect was significant; if yes, it represented the complementary mediating effect; otherwise, it represented competition. According to Table 1, the knowledge base has a complementary mediating effect between R&D intensity pre-M&A and the learning performance of enterprises (a*b*c = 0.467*0.442*0.276 > 0), confirming H1.

**TABLE 1:** Test of moderating effect of cultural and cognitive differences in learning mechanism 1 (*N* = 240).

Path	Coefficient	*T*-value
PRD→KB	0.467***	8.201
KB→LP	0.442***	8.691
PRD→LP	0.276***	4.674
PRD→KB→LP	0.206***	5.875
CoD→LP	−0.132*	2.634
CuD→LP	−0.085*	1.962
CoD*KB→LP	−0.132*	2.120
CuD*KB→LP	0.125*	2.009

PRD, represents R&D intensity pre-M&A; KB, represents knowledge base; CoD, represents cognitive difference; CuD, represents cultural difference; and LP, represents learning performance. *p < 0.05 (two-tailed); ***p < 0.01 (two-tailed).

#### Moderating Effects of Cultural and Cognitive Differences

As can be seen from the test results in Table 1, cognitive difference, a moderating variable, has a significant negative effect on enterprise learning performance (CoD significant nega = -0.132, *p* < 0.05). In addition, cultural differences have a significant negative effect on enterprise learning performance (CoDral differences ha= -0.085, *p* < 0.05), and the test of the moderating effect depends mainly on whether the product of the independent and moderating variables is significant. The product of cultural difference and knowledge base (CuD*KB) has a significant positive effect on the enterprise learning performance (CuD*KBear coefficient a = 0.125, *p* < 0.05), confirming H4. In addition, the product term of cognitive difference and knowledge base (CoD*KB) has a significant negative effect on the enterprise learning performance (CoD*KBear coefficient a = -0.132, *p* < 0.05), confirming H6.

### Hypothesis Test: Moderating Effect of Cultural and Cognitive Differences in Learning Mechanism 2

To test the effect of R&D intensity pre-M&A on learning performance, the mediating effect of R&D intensity post-M&A between the two, and the moderating effect of cultural and cognitive differences, this study set up a model according to the theoretical hypothesis and tested the path coefficient of the relationship between various variables in the theoretical hypothesis and the interpretation degree, *R*^2^ of the model through PLS-SEM and *t*-value significance analysis.

#### Mediating Effect of R&D Intensity Post-M&A

**FIGURE 3:**
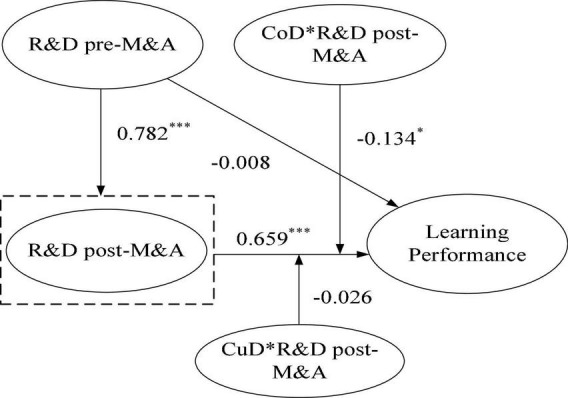
Path analysis results of learning mechanism 2. *p < 0.05 (two-tailed); ***p < 0.01 (two-tailed).

Figure 3 reports the test results of Learning Mechanism 2. We tested the three-step process of the mediating effect test. Specifically, (1) we checked whether the indirect effect of a (coefficient of PRD relat* b (coefficient of ARDthe indirect effect of a (coefficient of PRD relationship between there was no mediating effect; (2) we checked whether the coefficient c of the direct effect PRD→LP was significant; if no, it represented a full mediating effect; if yes, further observation was required; and (3) we checked whether a (coefficient of PRD→ARD) * b (coefficient of ARDthe c* c (coefficient of PRDthP) was positive; if it was positive, the mediating effect was complementary; otherwise, it had a competitive mediating effect.

The test was conducted according to a three-step process (refer to Figure 3). First, we checked the significance of the indirect effect (a*b) and analyzed whether there was a mediating effect. Table 2 reports that R&D intensity pre-M&A has a significant impact on R&D intensity post-M&A (PRD→ARD coefficient a = 0.782, *p* < 0.001), R&D intensity post-M&A has a significant impact on learning performance (ARD→ LP coefficient *b* = 0.659, *p* < 0.001) and an indirect effect a * b = 0.782 * 0.659 = 0.515 (*p* < 0.001), indicating a mediating effect. Second, after confirming the mediating effect, we checked whether the direct effect (c) was significant and judged whether there was a full mediating effect. Table 2 reports that the impact of R&D intensity pre-M&A has no significant effect on the learning performance of merging enterprises (the coefficient of PRDct on = -0.008, *p* > 0.05), indicating that R&D post-M&A has a full mediating effect between R&D intensity pre-M&A and the learning performance of enterprises, confirming H2.

**TABLE 2:** Test of moderating effect of cultural and cognitive differences in Learning Mechanism 2 (*N* = 240).

Path	Coefficient	*T*-value
PRD→ARD	0.782***	23.494
ARD→LP	0.659***	10.537
PRD→LP	–0.008	0.119
PRD→ARD→LP	0.515***	9.369
CoD→LP	−0.115*	2.394
CoD*ARD→LP	−0.134*	2.179
CuD→LP	–0.087	1.729
CuD*ARD→LP	–0.026	0.551

CoD, represents cognitive difference; CuD, represents cultural difference; LP, represents learning performance; ARD, represents R&D intensity post-M&A; PRD, represents R&D intensity pre-M&A. *p < 0.05 (two-tailed); ***p < 0.01 (two-tailed).

#### Moderating Effects of Cultural Differences and Cognitive Differences

Hypothesis 7 proposes that the cognitive difference negatively moderates the R&D intensity post-M&A and learning performance. Table 2 shows the results that the cognitive difference is negatively and significantly associated with learning performance (the coefficient of CoDurthe = -0.115, *p* < 0.05). We added the interaction terms into Mechanism 2 to examine the moderating effects. Our results show that the presence of R&D intensity post-M&A has no significant impact on the learning performance; hence, the results of Learning Mechanism 2 support H7.

### Hypothesis Test: Moderating Effect of Cultural and Cognitive Differences in Learning Mechanism 3

To test the impact of R&D intensity pre-M&A on learning performance, the double mediating effect of knowledge base and R&D intensity post-M&A between the two and the moderating effect of cultural and cognitive differences, this study set up a model according to the theoretical hypothesis and tested the path coefficient of the relationship between variables in the theoretical hypothesis and *R*^2^, the interpretation degree of the model through PLS-SEM, and *t*-value significance analysis.

**FIGURE 4:**
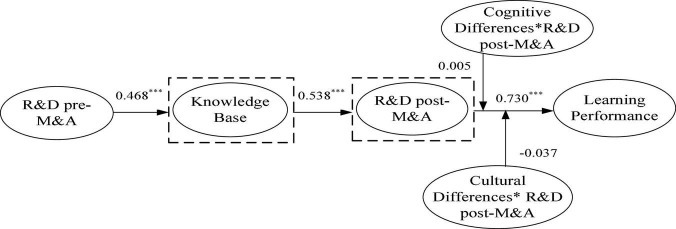
Path analysis results of learning mechanism 3. *p < 0.05 (two-tailed); ***p < 0.01 (two-tailed).

*The sequential mediating effect of knowledge base and R&D intensity post-M&A.* Hypothesis 3 proposes that knowledge base and R&D intensity pre-M&A have a sequential mediating effect on the learning performance. Figure 4 reports the results of Learning Mechanism 3. To test Hypothesis 3, we tested the mediation effect according to the above three-step process. As shown in Learning Mechanism 3, the indirect effect, R&D intensity pre-M&Are-tensity base→R&D intensity post-M&Aost-ensiise learning performance (PRDted the mediation effect according *p* < 0.001), is significant, indicating a mediating effect. Table 3 shows that the direct effect of R&D intensity pre-M&A on the learning performance of enterprises is not significant (PRDance of enterprdia= -0.113, *p* > 0.05), indicating that knowledge base and R&D intensity post-M&A play a full mediating effect between R&D intensity pre-M&A and the learning performance of enterprises; thus, Hypothesis 3 is strongly supported.

**TABLE 3:** Test of moderating effect of cultural and cognitive differences in Learning Mechanism 3 (*N* = 240).

Path	Coefficient	*T*-value
PRD→KB	0.468***	7.815
KB→ARD	0.538***	9.474
ARD→LP	0.730***	11.494
PRD→LP	–0.113	1.502
CoD→LP	–0.052	0.898
CuD→LP	–0.037	0.725
CoD*ARD→LP	0.005	0.101
CuD*ARD→LP	–0.037	0.725
PRD→KB→ARD→LP	0.184***	3.99

CoD, represents cognitive difference; CuD, represents cultural difference; LP, represents learning performance; ARD, represents R&D intensity post-M&A; PRD, represents R&D intensity pre-M&A. *p < 0.05 (two-tailed); ***p < 0.01 (two-tailed).

#### Moderating Effects of Cultural Differences and Cognitive Differences

We added the interaction terms into the model to examine the moderating effects. Our results show that the interaction terms lose significance in Learning Mechanism 3 (CoD*ARDoDi coefficient *a* = -0.146, *p* < 0.05), which suggests that cognitive differences have no moderating effect on the two. In addition, the interaction term (CuD*PRD) of cultural differences and R&D intensity pre-M&A has no significant effect on the learning performance of enterprises (*a* = 0.037, *p* > 0.05), indicating that cultural difference has no moderating effect on the two; hence, the moderating effect was not significant in this model.

## Discussion and Conclusion

We conducted a theoretical analysis of the organizational learning mechanism of technology-acquiring cross-border M&A, thus constructing a theoretical model of the learning mechanism of technology-acquiring cross-border M&A from EMEs with the interaction of technical capabilities, cultural differences, and cognitive differences. Based on an empirical study of 240 Chinese, technology-acquiring cross-border M&A enterprises, this study further clarified the mechanisms of the effect of cultural differences and cognitive differences on the organizational learning of cross-border M&A.

First, in the mode of technology-acquiring cross-border M&A, the knowledge base has a complementary mediating effect on the relationship between the R&D intensity of pre-M&A and learning performance, cultural differences positively moderate the relationship between the knowledge base and learning performance, while cognitive difference negatively moderates the relationship between the knowledge base and learning performance. In the process of organizational learning of cross-border M&A of EMEs, many M&A enterprises will inevitably encounter huge cultural and cognitive differences. The cultural and cognitive habits of different countries are not only an important source of “outsider disadvantage” of EMEs but also an important factor affecting the organizational learning of enterprises in reverse cross-border M&A. The greater the cultural and cognitive differences, the more difficult it is for EMEs to transfer technology; for example, Haier has a strong knowledge base before M&A. Although the cultural differences between the acquired enterprises are large, the cognitive differences between Haier and the M&A party are small. In 2001, after acquiring the Italian company Meneghetti, Haier made full use of the small cognitive differences between the two sides to implement cooperative R&D and innovation, so as to achieve better learning performance.

Second, in the mode of technology-acquiring cross-border M&A, the R&D intensity of post-M&A has a full mediating effect on the relationship between R&D intensity of pre-M&A and learning performance of enterprises, and cognitive difference negatively moderates the relationship between R&D intensity of post-M&A and learning performance of enterprises. As the technological capability of enterprises in emerging economies is often relatively weak, in the face of the rapidly changing international market and technological environment, enterprises intend to realize technological learning through cross-border M&A. Post-merger R&D is an important way for enterprises to improve learning and performance. The cross-cultural and cognitive differences between the two sides of M&A often bring great challenges to the organizational learning of enterprises. For instance, in February 2013, CIMC completed the full acquisition of CIMC Raffles Offshore (Singapore) Limited due to the small cognitive difference between China and Singapore and finalized the business and management integration of CIMC Raffles in only 4 years. After the M&A, CIMC has continuously strengthened its R&D investment, continuously improving the R&D capacity of a single technology. The CIMC combines its technical, management, and financial advantages with Raffles’ technical advantages to achieve better learning performance.

Finally, the knowledge base and R&D intensity of post-M&A have a sequential mediating effect between R&D intensity of pre-M&A and learning performance, while cultural and cultural differences have no significant moderating effect. M&A enterprises have a strong knowledge base, and the R&D intensity before and after M&A is very high, which indicates that the enterprises rely on their own strong technical ability, overcome the barriers of cultural and cognitive differences, integrate the advanced R&D network of the M&A enterprises with their own R&D network and not only make use of the advanced technology of the host country but also actively explore and expand new technology. Technical capability is an important driving force of enterprise learning performance, and the innovation of learning based on the technology-acquiring cross-border M&A of EMEs comes from two kinds of driving forces: on the one hand, through exploitation learning of the external technology and knowledge of acquired enterprises in developed countries; on the other hand, through exploratory learning of new technical knowledge by effectively combining the external technical knowledge of acquiring enterprises and the internal knowledge of acquired enterprises. The model confirms that when an enterprise has a particularly strong technical capability—for instance, a strong technical foundation and large R&D investment before and after the merger—it can overcome cultural and cognitive differences. Let us consider Wanxiang as an example. Although the cultural and cognitive differences between the Chinese and American enterprises are significant, since 2001, Wanxiang has successfully acquired many auto parts and new energy vehicle industry enterprises, such as UAI companies. Most acquired enterprises have the key technologies or R&D capabilities required by Wanxiang and are highly related to its technology. Therefore, Wanxiang has formed a strong knowledge base through exploitation and exploratory learning with years of high-intensity R&D investment and strong innovation ability. In the strategic integration after M&A, Wanxiang desalinated the enterprise’s home country color, followed the American cultural and cognitive habits and legal system, conducted localized governance, successfully leveraged and integrated global innovation resources, and reverse learned and absorbed advanced technology and knowledge.

### Theoretical Contributions

The research contribution of this study mainly lies in the following aspects.

First, based on the integration theory of cultural differences (Bauer et al., 2016; Guiso et al., 2016; Gu and Meng, 2021), the cognitive difference (Ager, 2011) and technical ability (Viegas Pires, 2013), this study expands the understanding of the learning mechanism of technology-acquiring cross-border M&A in the emerging markets (Stahl and Voigt, 2008). Although there are many theoretical and empirical studies on cross-border M&A, they are mainly based on a single perspective, such as technical capability. However, the learning mechanism of technology-acquiring cross-border M&A in the emerging markets is the result of a combination of multiple factors in a complex situation. Compared to previous studies, this study conducts an in-depth analysis of the learning mechanism of EMEs’ technology-acquiring cross-border M&A from the perspective of integration theory. Based on the integration theory of cultural differences, cognitive differences, and technical abilities, this study expands the theoretical research perspective in this field.

Second, the study demonstrates, from both theoretical and empirical aspects, three kinds of reverse learning paths for enterprises to achieve higher learning performance in the context of cross-cultural and cognitive differences between emerging and developed markets. The PLS-SEM model was introduced into the theoretical model to explore how cultural and cognitive differences interact with technical capabilities in the process of technology-acquiring cross-border M&A. This paper reveals the black box of the learning mechanism of technology-acquiring cross-border M&A by emerging markets and further describes the learning mechanism under the influence of cultural and cognitive differences.

Third, previous studies were mainly conducted from a single dimension of technical ability (Chen et al., 2009; Benitez-Amado and Ray, 2012; Feng et al., 2018) or cross-cultural differences (Forstmann, 1998; Zhu and Huang, 2007), and few studies have compared multiple factors (Zhang et al., 2018). No relevant dialogue exists regarding which dimension has a more important impact on the innovation performance of technology-acquiring cross-border M&A in emerging markets, and no consistent conclusion has been reached. We find that compared to cultural differences, cognitive differences have a more significant impact on the learning performance of technology-acquiring cross-border M&A; however, when EMEs have strong technical capabilities, they can overcome the barriers of cultural and cognitive differences and achieve better learning performance. The conclusion of this study not only contributes to previous theoretical research but also has important implications for enterprises to carry out technology-acquiring cross-border M&A in emerging markets.

### Practical Implications

In the process of cross-border M&A, EMEs face the challenges of large cognitive differences, cultural differences, and technological gaps between the home and host countries. How should EMEs improve their knowledge base to cross-cultural and cognitive differences and achieve better organizational learning performance? First, they can do so by strengthening the enterprise’s own R&D investment, so as to continuously improve the R&D ability of single technology or some individual products and form its own strong knowledge base. Second, M&A enterprises can build a cooperative R&D platform with the acquired enterprises through joint venture subsidiaries or joint R&D projects and further improve their overall and systematic R&D capacity through the R&D investment after M&A, so as to achieve better organizational learning performance. In this process, the R&D personnel of the M&A enterprise should cooperate with the R&D team of the acquired enterprises, continuously strengthen cooperation and communication in the R&D process, improve the knowledge base of the enterprise, enhance the organizational cultural identity of both sides, and reduce their cognitive differences, so as to promote the M&A enterprise to better absorb the advanced technology of the acquired enterprises.

Third, the EMEs should use more platforms, such as joint venture subsidiaries, joint R&D projects, or cooperative R&D platforms, to be more conducive to decreasing the cultural and cognitive differences. However, it is necessary for the acquirer to actively cooperate with the acquire in the fields of technology and R&D. When facing the different challenges of cross-cultural differences and technology gaps, EMEs should use joint venture subsidiaries, joint R&D projects, or cooperative R&D platforms between key domestic enterprises and first-mover enterprises in developed countries to improve technology acquisition. It is worth noting that the smaller the cognitive differences between key enterprises and first-mover enterprises, such as the smaller the ideological and oral differences between the two sides, the more conducive it is to the cooperation and communication between the R&D personnel of the focal company and the R&D team of the pioneer company in the process of joint R&D, and the more conducive it is to the absorption of the advanced technology by the pioneer company.

Fourth, the EMEs should strengthen the cooperation with the acquired enterprises in the fields of strategic cooperation, human resource management, and organizational structure, whenever before or after M&A. First, before M&A, it is necessary for EMEs to actively cooperate with the acquired enterprises in the fields of technology and R&D—a measure more conducive to enhancing mutual understanding and cognition. Technology exchange and cooperation are not only a necessary means but also an “olive branch” that provides a cognitive basis and starting point for the all-round strategic cooperation after M&A. Second, after M&A, when enterprises in emerging economies face the challenges of cross-cultural and cognitive differences in the process of integration, EMEs should integrate background supporting activities, such as human resource management, to support cross-cultural management. Enterprises should increase international job rotation opportunities, absorb talent outside the industry in a more open way, and establish a cross-cultural talent team. In terms of organizational structure, M&A enterprises should encourage mutual cooperation and establish cross-cultural and cross-organizational management policies and systems.

### Limitations and Future Directions

This study has some limitations and directions for future research. A limitation concerns the study’s sample. This study was conducted in China, an emerging market; as such, Chinese cross-border M&A are more likely to acquire technology from the developed countries and may learn differently from other emerging markets. Furthermore, cross-cultural and cognitive differences may vary across the emerging markets and limiting the sample to China may thus bias the results to some extent. Scholars should determine whether this conclusion holds in other emerging markets to further confirm the generalizability.

## Data Availability Statement

The original contributions presented in the study are included in the article/supplementary material, further inquiries can be directed to the corresponding author/s.

## Author Contributions

KC and XC participated in the design, data collection, drafting of the first version, and revision of the article. RZ participated in drafting the first vision. All authors have contributed to the manuscript and approved the submitted version.

## Conflict of Interest

The authors declare that the research was conducted in the absence of any commercial or financial relationships that could be construed as a potential conflict of interest.

## Publisher’s Note

All claims expressed in this article are solely those of the authors and do not necessarily represent those of their affiliated organizations, or those of the publisher, the editors and the reviewers. Any product that may be evaluated in this article, or claim that may be made by its manufacturer, is not guaranteed or endorsed by the publisher.
